# Data science approach to stock prices forecasting in Indonesia during Covid-19 using Long Short-Term Memory (LSTM)

**DOI:** 10.1186/s40537-021-00430-0

**Published:** 2021-03-11

**Authors:** Widodo Budiharto

**Affiliations:** Computer Science Department, School of Computer Science, Bina Nusantara University, Jakarta, 11480 Indonesia

**Keywords:** Data science, LSTM, Forecasting, Stock market, Finance, Deep learning

## Abstract

**Background:**

Stock market process is full of uncertainty; hence stock prices forecasting very important in finance and business. For stockbrokers, understanding trends and supported by prediction software for forecasting is very important for decision making. This paper proposes a data science model for stock prices forecasting in Indonesian exchange based on the statistical computing based on R language and Long Short-Term Memory (LSTM).

**Findings:**

The first Covid-19 (Coronavirus disease-19) confirmed case in Indonesia is on 2 March 2020. After that, the composite stock price index has plunged 28% since the start of the year and the share prices of cigarette producers and banks in the midst of the corona pandemic reached their lowest value on March 24, 2020. We use the big data from Bank of Central Asia (BCA) and Bank of Mandiri from Indonesia obtained from Yahoo finance. In our experiments, we visualize the data using data science and predict and simulate the important prices called Open, High, Low and Closing (OHLC) with various parameters.

**Conclusions:**

Based on the experiment, data science is very useful for visualization data and our proposed method using Long Short-Term Memory (LSTM) can be used as predictor in short term data with accuracy 94.57% comes from the short term (1 year) with high epoch in training phase rather than using 3 years training data.

## Introduction

Data science is a blend of various tools, algorithms, and machine learning principles with the goal to discover hidden patterns from the raw data. Using data science and forecasting method, we can see get many insight such as the financial health of a company. A forecasting algorithm is an information process that seeks to predict future values based on past and present data. The forecasting is so important because prediction of future events is a critical input into many types of planning and decision-making processes such as finance, industrial process control risk management [1].

Time series analysis has significance in financial analytic and forecasting and it can be utilized in any field. In finance, time series analysis is used for financial forecasting such as stock prices, assets, and commodities. Stock is the most volatile investment with high risk, but with high return to investors if carefully managed in their portfolio. In managing stocks, information on their prices is of utmost importance. Capital markets are markets for buying and selling equity and debt instruments, it also has activities related to public offering and trade of stock and issuance stock of public company. Stock exchanges are considered major players in financial sectors of many countries included Indonesia. Stockbrokers, who execute stock trade, use technical, fundamental or time series analysis in trying to predict stock prices, so as to advise client [2].

The capital market on the Indonesia Stock Exchange (IDX) [3] in 2020 is in an uncertain condition since the outbreak of the corona virus (Covid-19) in Indonesia. Many issuers' shares have dropped, including state companies, aka state-owned enterprises. There are at least 10 state-owned enterprises (BUMN) shares whose prices have dropped considerably since the end of 2019 until now, such as the share price of PT Adhi Karya Tbk (ADHI) which experienced a drastic drop in just the last 60 trading days. As well as PT Semen Indonesia Tbk (SMGR) [4]. In the midst of the COVID-19 pandemic and the dynamics of the global financial market during Semester I 2020, the Jakarta Composite Index (JCI) and the majority of global stock index reference indexes experienced a significant decline. As of August 7, 2020, JCI was still closed in the red zone with − 18.34%. The IDX suspended short selling as the Jakarta Composite Index (JCI) was in a free-fall, continuing its losses since the start of 2020. The IDX believed the stock market correction in Indonesia was mirroring similar losses around the world over fears of the coronavirus pandemic.

Today, artificial intelligence (AI) is a thriving field with many practical applications and active research topics. Many researcher on data science and deep learning try to predict stock prices forecasting such as using LSTM [5–7]. This paper proposes an efficient, simple model and algorithm for big data analysis using R language and LSTM for stock forecasting with improvement and innovation in selecting only short-term data for training phase and able to gives future prediction value and of course should be very useful for stock prices prediction in Indonesia. The section of paper consists of introduction, literature review, proposed method, result and discussion and conclusion section.

## Literature review

### Stock prices forecasting

Predicting stock prices is very important for finance practitioners to best allocate their assets and to academics to build better and more accurate asset pricing models. Predicting stock returns gives crucial implications about market efficiency. Prediction of future movement of stock prices has always been a challenging task for the researchers. In fact, investors are highly interested in the research area of stock price prediction. Time series forecasting analyzes past data and projects estimates of future data values. Basically, this method attempts to model a nonlinear function by a recurrence relation derived from past values. A comparative study of LSTM and Deep Neural Network for Stock Market Forecasting has been conducted by [8]. The Efficient Market Hypothesis (EMH) states that at any time, the price of a share fully captures all known information about the share. Since all known information is used optimally by market participants, price variations are random, as new information occurs randomly. Thus, share prices perform a "random walk", and it is not possible for an investor to beat the market. Many researchers propose a model for stock price forecasting, such as [9] proposed a model for prediction using neural network to discover nonlinear relationships in input data makes them ideal for modeling nonlinear dynamic systems such as the stock market.

Another method for stock prices prediction is using ARIMA (Autoregressive Integrated Moving Average). In an ARIMA model, the future value of a variable is supposed to be a linear combination of past values and past errors. Assumptions of ARIMA model that data should be stationary—by stationary it means that the properties of the series do not depend on the time when it is captured. A white noise series and series with cyclic behavior can also be considered as stationary series [10, 11]. Based on our previous result, LSTM is better compared with ARIMA model [7].

### Dataset of stock prices from Yahoo Finance

**Fig. 1:**
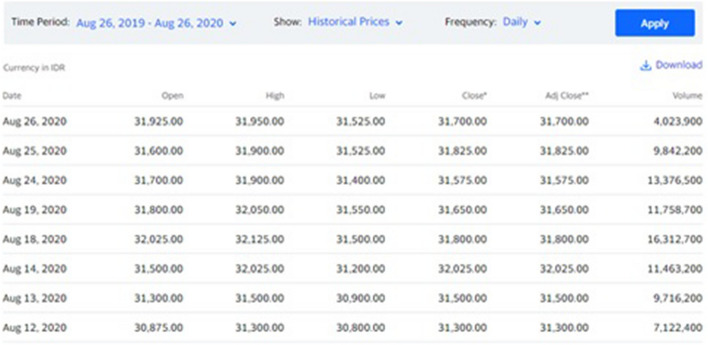
Example of datasheet from Yahoo Finance [10]

Yahoo Finance is the largest business and financial news site in the world, with unrivaled access to data, insights, and content. The example of datasheet from Yahoo Finance is shown in Fig. 1.

**Fig. 2:**
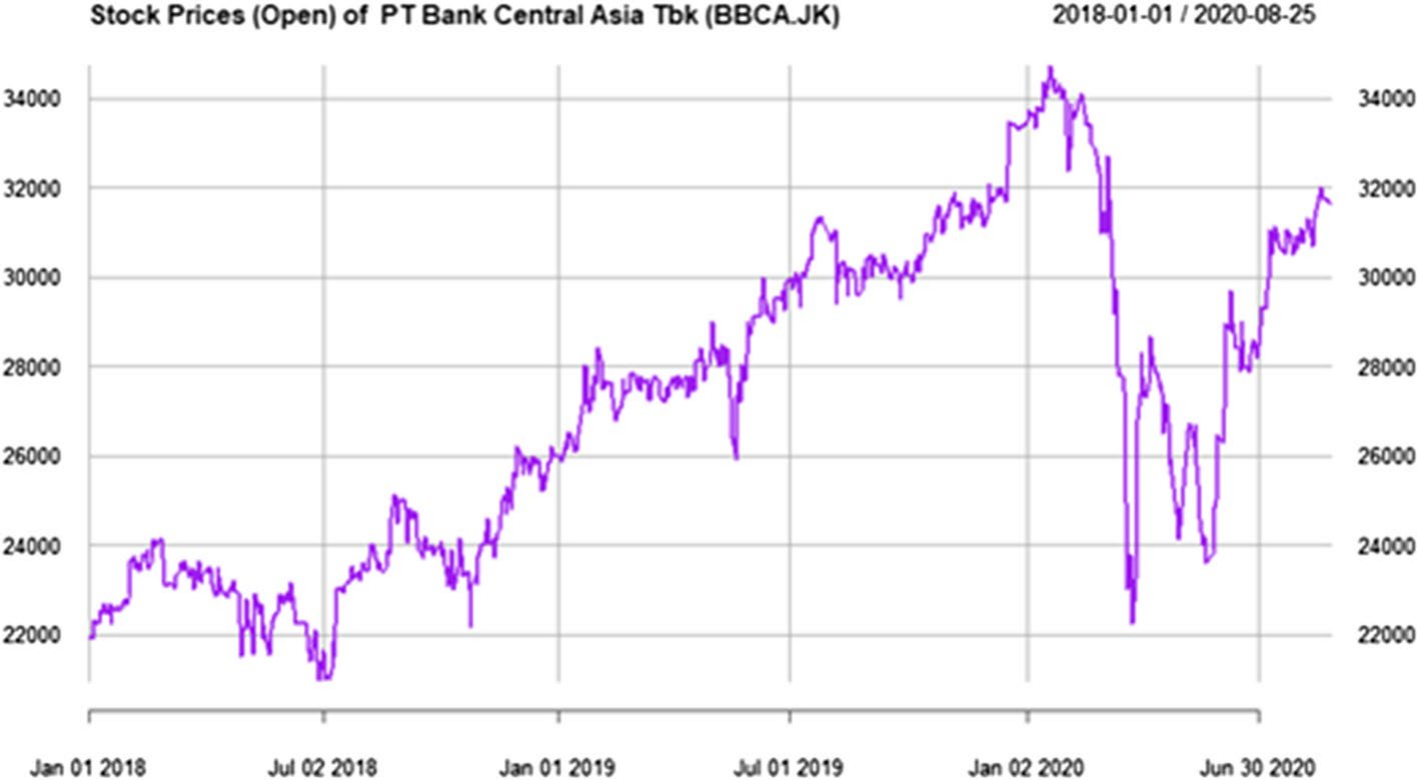
Example of data visualization using data science approach

Data science approach focus on how to display data that easily understood by the decision maker. Data visualization is an important feature in data science approach, as shown in Fig. 2, we can see data between 2018 and 2020, that the best stock prices condition of Bank BCA at the beginning of year 2020.

### Artificial intelligence (AI) for stock prices prediction

**Fig. 3:**
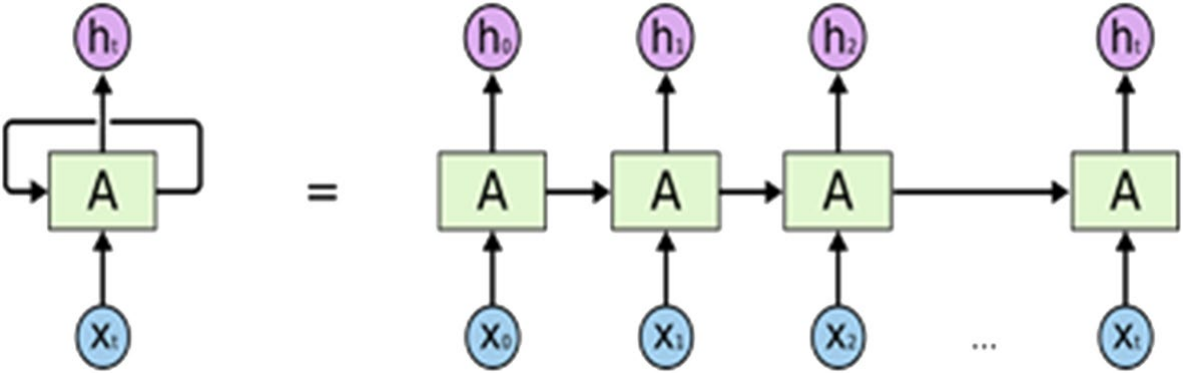
The basic model of Recurrent neural networks (RNN) [6]

Sequence prediction problems have been around for a long time especially in financial markets. LSTM built from the Recurrent Neural Network (RNN). In the figure shown, a chunk of neural network **A**, looks at some input *x*_*i*_ and outputs a value *h*_*i*_*.* A loop allows information to be passed from one step of the network to the next as shown in Fig. 3.

A typical LSTM network is comprised of different memory blocks called cells. There are two states that are being transferred to the next cell; the cell state and the hidden state. The memory blocks are responsible for remembering things and manipulations to this memory is done through three major mechanisms, called gates. LSTMs are particularly well suited to time-series prediction because they can “learn” and “remember” in long-term memory things like market regimes, whereas short-term memory and good interaction with look back windows (and even time-irregular data or large steps between significant events) leads to solid performance in short-term trend prediction [12].

## Proposed method

**Fig. 4:**
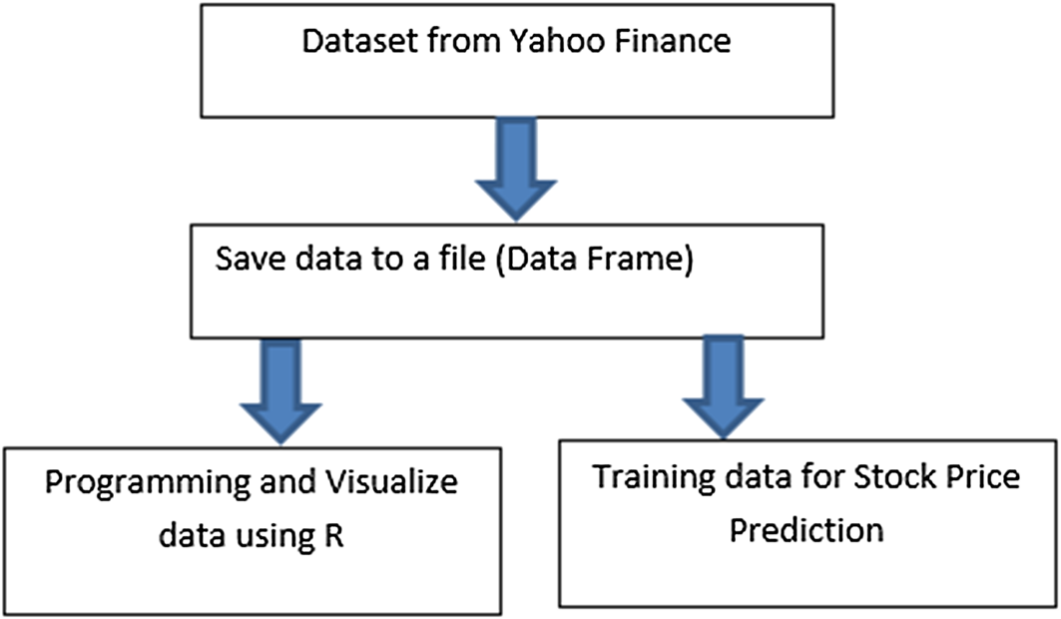
The model for stock prices prediction using big data from Yahoo Finance

The flow of data science approach for data visualization and stock prices prediction based on big data from Yahoo is shown in Fig. 4.

Considering the complexity of financial time series, combining deep learning with concept of financial market prediction is regarded as one of the most charming topics [13, 14]. Based on that idea, we propose the algorithm for predicting of future values and the RNN model that has LSTM [15, 16]. We use values from the very beginning in the first sliding window to predict the price *p* in the following window *W*_*t*+1_:

1 $$W_{t + 1} = \left( {p_{(t + 1)w} ,p_{(t + 1)w + 1} , \ldots ,p_{( + 2)w - 1} } \right).$$

**Fig. 5:**
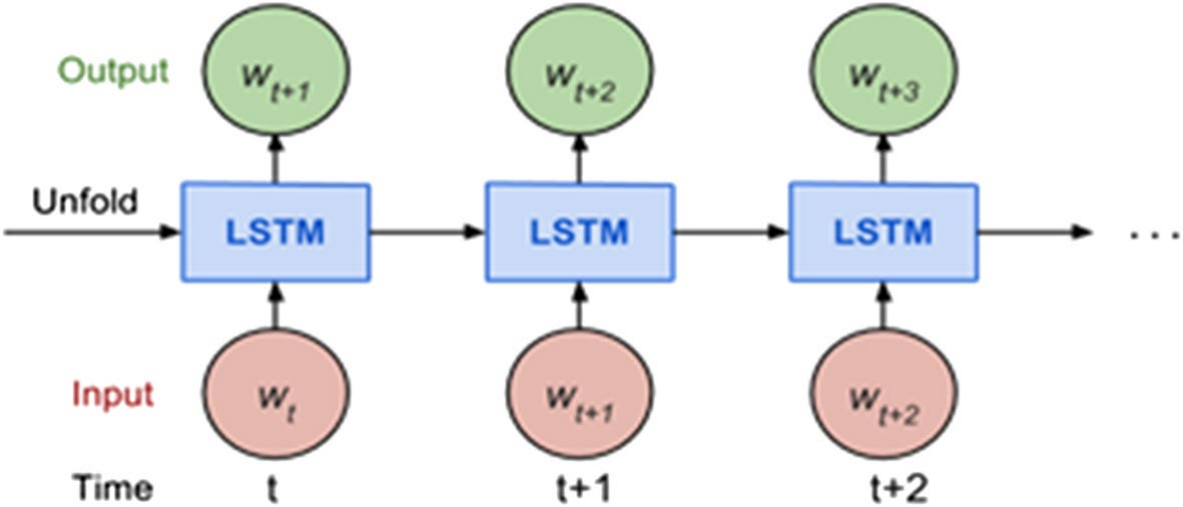
The Rnn model for stock prices prediction

Figure 5 shows our model for stock prices prediction.

The efficient algorithm based on Tensorflow and LSTM for prediction of stock prices is shown in Algorithm 1.
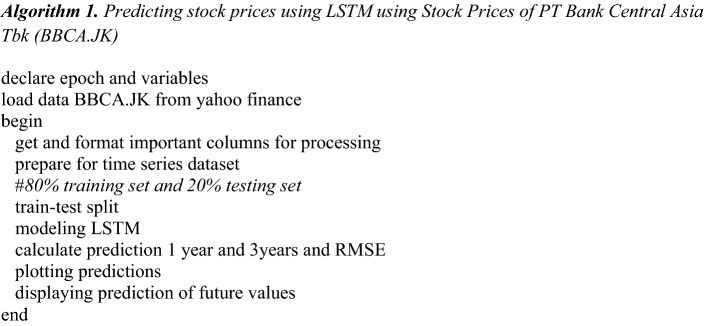


## Result and discussion

**Fig. 6:**
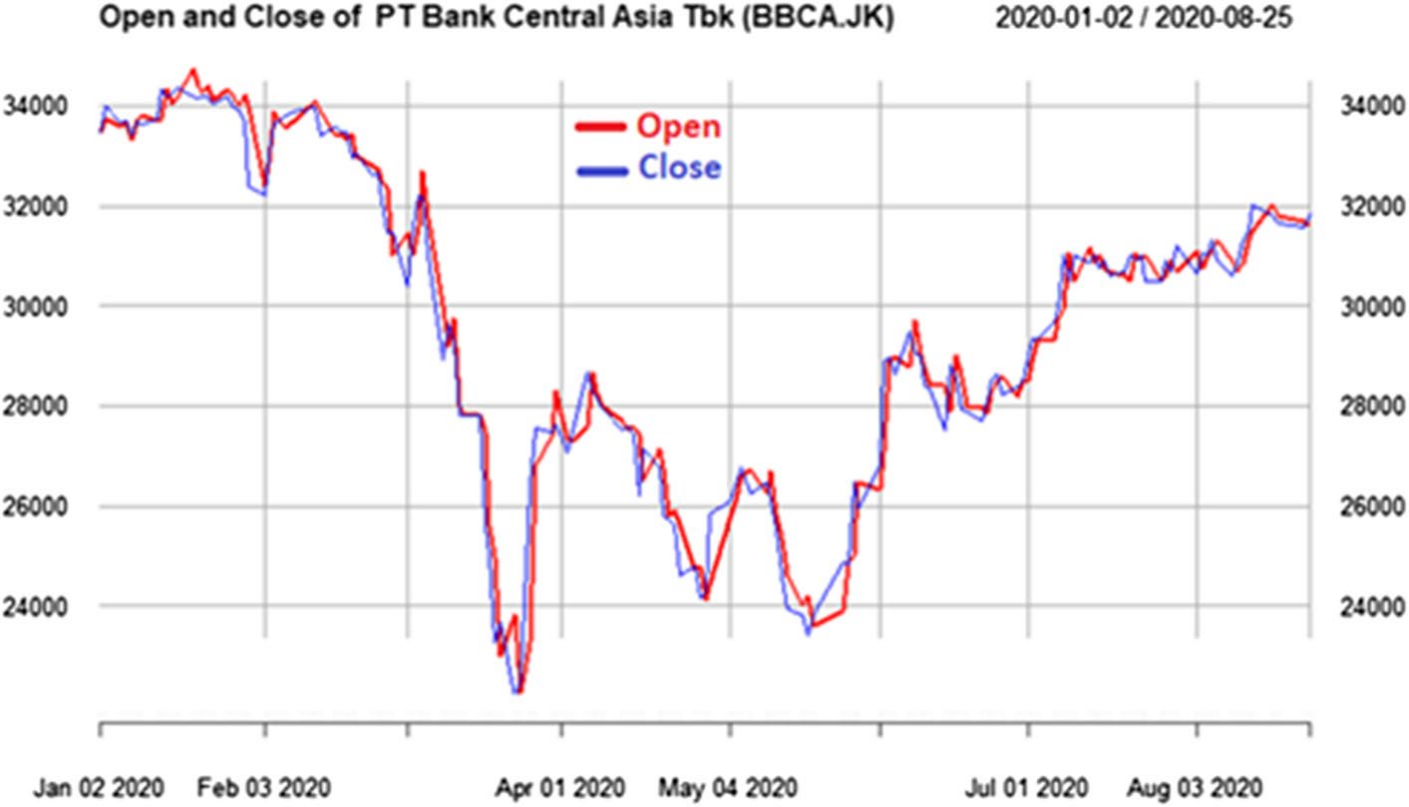
Stock prices in 2020 from PT Bank Central Asia Tbk (BBCA.JK), the lowest price in 24 March 2020

**Fig. 7:**
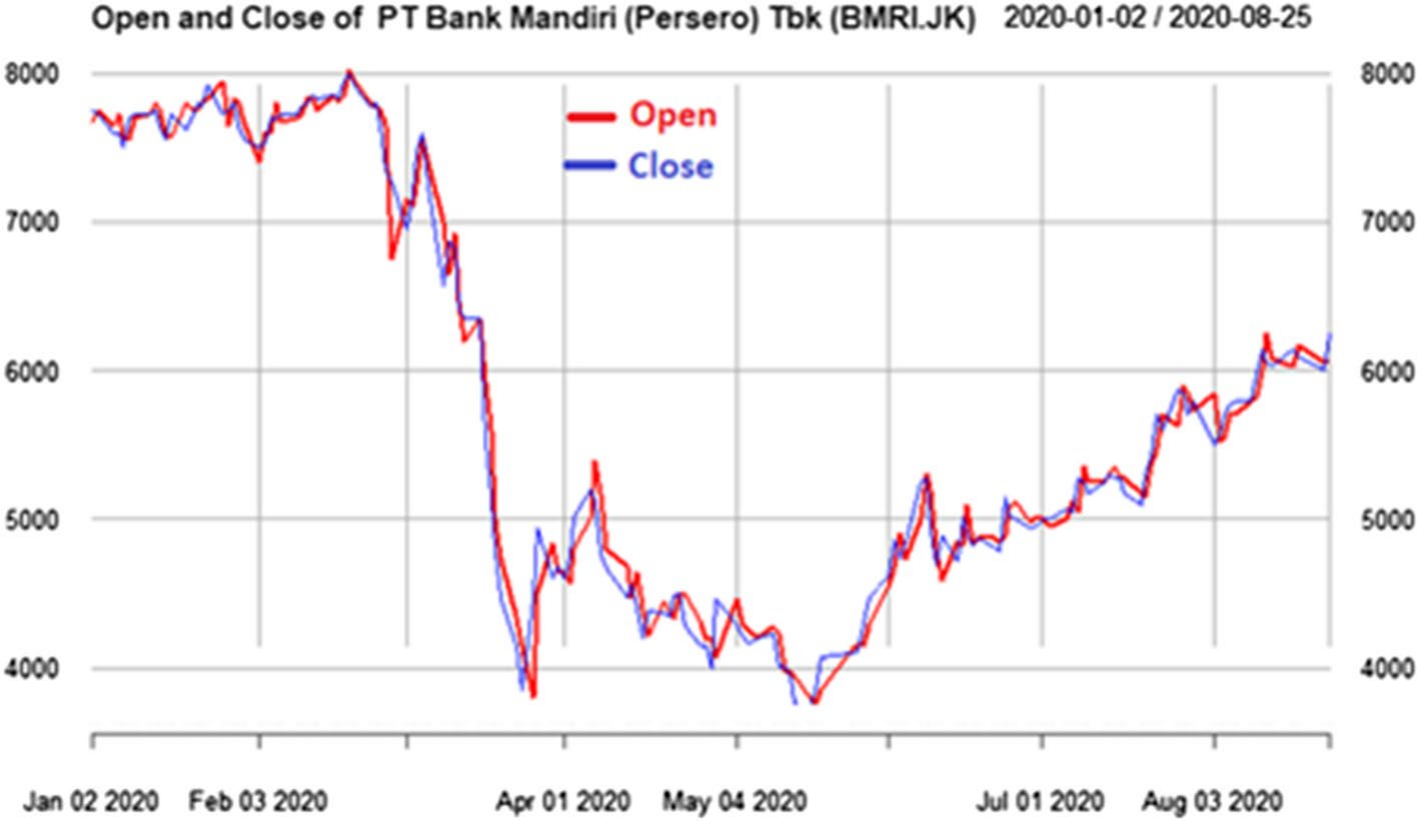
Stock prices in 2020 from PT Bank Mandiri (Persero) Tbk(BMRI.JK), the lowest price also in 24 March 2020

Based on data science approach, we can have insight Example of Stock Prices of PT Bank Central Asia Tbk (BBCA.JK) and PT Bank Mandiri from Indonesia at Yahoo finance are shown in Figs. 6 and 7. The first Covid-19 confirmed case in Indonesia is on 2 March 2020. After that, the composite stock price index has plunged 28% since the start of the year 2020, the share prices of cigarette producers and banks in the midst of the corona pandemic reached their lowest value on March 24, 2020 easily can be seen from data visualization.

**Fig. 8:**
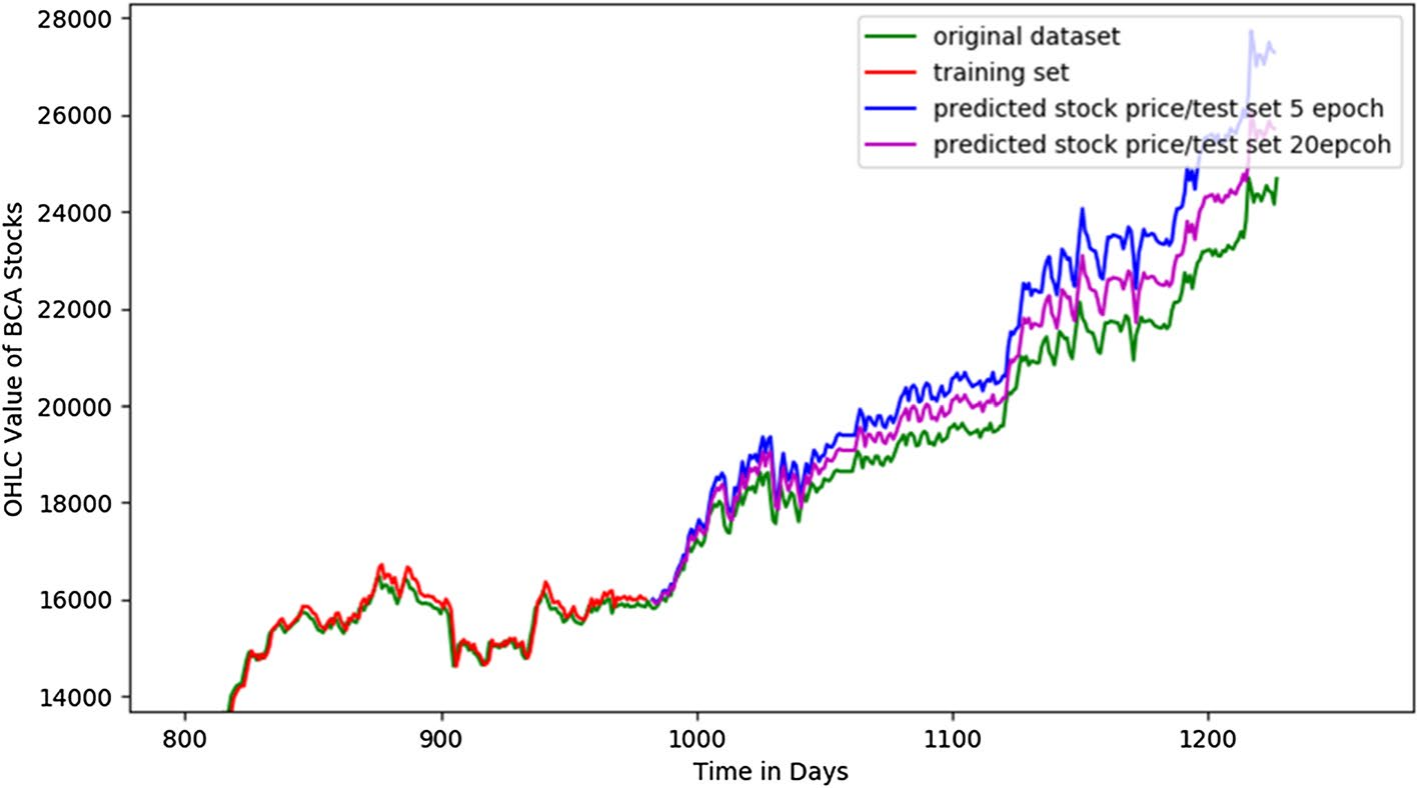
Detailed result of prediction based on the 80% training set and 20% testing set. It shows the significant accuracy from 5 to 20 epochs

**Fig. 9:**
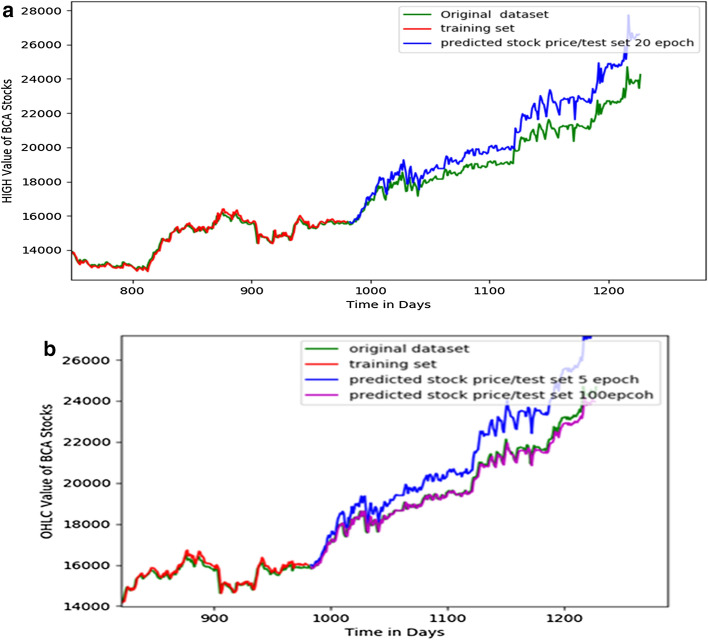
Result of prediction of high price using 20 epochs (**a**) and OHLC price using 5 and 100 epochs. Prediction using 100 epochs is better than 5 epochs (**b**)

We developed LSTM program using Python and Tensorflow for stock prices prediction [12]. We use 80% for training data and 20% for testing data, and the result shown in Figs. 8, 9.

We compare result of the experiment by varying epoch and historical data between 1 and 3 years as shown in Table 1. It shows that the best prediction using 1 year data with the best accuracy 94.59% at 100 epoch. Epoch is one of the best methods to compare various data for forecasting. For analyzing the efficiency of the system we are used the Root Mean Square Error (RMSE). Comparing with other research for stock price forecasting, our method is better (usually neural network method only about 90% accuracy) [17].

**Table 1:** Result of experiment with various historical data and epoch (with epoch 100 in 1 year, the best accuracy 94.59% is reached)

Historical data	OHLC value			High value		
	Epochs					
	5	20	50	20	50	100
Test RMSE value						
1 year	537.07	335,33	257.42	565.18	394.76	205.65
	Prediction value (next day)		25,545.20	25,341.33	25,343.35	25,344.05
	Accuracy		93.83%	94.57%	94.58%	*94.59%*
3 years	1023.43	1022.43	1023.45	929.80	193.30	193.31
	Prediction value (next day)		30,619.65	28,690.60	27,094.52	27,092.09
	Accuracy		78.28%	83.54%	88.46%	88.47%

## Conclusion

This paper develops a model and program for stock prices prediction using data from Yahoo finance. Efficient and accurate prediction systems for stock prices help traders, investors, and analyst by providing supportive information like the future direction of the stock market. We found that for LSTM, it should use short term historical data for the best accuracy. Historical data using 1 year is the best compared with 3 years and 5 years data. Deep learning technology is expanding the options available to data scientists to solve interesting problems with high accuracy. LSTM also superior in short term data until 94.59% as shown in Table 1. Data science approach proved to be used easily for decision maker and companies to get better view of stock prices or their financial health condition. For future work, we will improve our method using recent deep learning methods. At the end of 2020, amid the COVID-19 Pandemic, the number of Indonesia Capital Market investors continues to increase rapidly. The number of Indonesia Capital Market investors, according to data recorded in KSEI as of December 29, 2020, increased by more than 50% to 3,871,248 from the previous 2,484,354 at the end of 2019. So, we have to optimize to solve the pandemic with empowering the business at all sectors.

## Data Availability

Not applicable.

## Abbreviations

BUMN: Badan Usaha Milik Negara *(stated-owned enterprises)*
IHSG: Index Harga Saham Gabungan (composite stock price index)
